# SETD6 controls the expression of estrogen-responsive genes and proliferation of breast carcinoma cells

**DOI:** 10.4161/epi.28864

**Published:** 2014-04-21

**Authors:** Daniel J O'Neill, Stuart Charles Williamson, Dhuha Alkharaif, Isabella Christina Mazzaro Monteiro, Marilyn Goudreault, Luke Gaughan, Craig N Robson, Anne-Claude Gingras, Olivier Binda

**Affiliations:** 1 Northern Institute for Cancer Research; Newcastle University; Newcastle upon Tyne, UK; 2 Lunenfeld-Tanenbaum Research Institute; Mount Sinai Hospital; Toronto, ON Canada; 3 Department of Molecular Genetics; University of Toronto; Toronto, ON Canada

**Keywords:** lysine methyltransferase, SETD6, estrogen receptor, cellular proliferation

## Abstract

The lysine methyltransferase SETD6 modifies the histone variant H2AZ, a key component of nuclear receptor-dependent transcription. Herein, we report the identification of several factors that associate with SETD6 and are implicated in nuclear hormone receptor signaling. Specifically, SETD6 associates with the estrogen receptor α (ERα), histone deacetylase HDAC1, metastasis protein MTA2, and the transcriptional co-activator TRRAP. Luciferase reporter assays identify SETD6 as a transcriptional repressor, in agreement with its association with HDAC1 and MTA2. However, SETD6 behaves as a co-activator of several estrogen-responsive genes, such as *PGR* and *TFF1*. Consistent with these results, silencing of SETD6 in several breast carcinoma cell lines induced cellular proliferation defects accompanied by enhanced expression of the cell cycle inhibitor *CDKN1A* and induction of apoptosis. Herein, we have identified several chromatin proteins that associate with SETD6 and described SETD6 as an essential factor for nuclear receptor signaling and cellular proliferation.

## Introduction

Transcription is regulated by the recruitment of chromatin remodeling and histone modifying activities at gene regulatory elements such as promoters and enhancers. Histone modifiers include several regulatory factors such as histone acetyltransferases (HAT), histone deacetylases (HDAC), lysine methyltransferases (KMT), and lysine demethylases (KDM). It is generally accepted that the acetyl group neutralizes the positive charge of the side chain of the lysine residues within the histone tails thereby diminishing the interaction with the negatively charged DNA,^1^^-^^3^ allowing an open chromatin conformation and accessibility to transcription factors. In addition, acetyl-lysines are recognized by small protein domains such as bromodomain.^4^ Similarly, methyl groups on lysines serve as docking sites for small protein domains that stabilize protein-protein interactions between histones and effector proteins.^4^ Lysines can be mono, di, or trimethylated and the consequences are site and state-specific. For example, H3K9^me1^ (histone H3 monomethylated on lysine 9), H3K4^me3^ (H3 trimethylated on lysine 4), and H3K36^me3^ are generally found in the vicinity of active promoters, while H3K9^me3^ and H3K27^me3^ are usually found at transcriptionally silenced promoters.^5^

Although histone lysine methylation was discovered over four decades ago,^6^^,^^7^ the first histone lysine methyltransferase was only identified 36 years later.^8^ The latest human genome annotation reveals approximately 66 potential KMTs containing a SET catalytic domain, a SET-like PR domain, or a 7-β-strand structure.^9^^,^^10^ The role of lysine methylation in human pathologies is highlighted by the increasing number of KMTs involved in the etiology of cancer,^11^^,^^12^ growth defects,^13^^,^^14^ and neurological disorders.^15^

The methyltransferase SETD6 was recently shown to monomethylate the histone H2A variant H2AZ on lysines 4 and 7 (H2AZK4^me1^K7^me1^) and demonstrated to be essential for the maintenance of self-renewal in embryonic stem cell.^16^ SETD6 was also found to methylate the RELA subunit of NFκB on lysine 310, thereby enhancing protein-protein interaction with the G9A-like methyltransferase EHMT1 (GLP) and resulting in transcriptional silencing of inflammation-regulated genes.^17^

Estrogen receptors have clear links to the etiology of breast cancer. Specifically, the estrogen receptor α (ERα or ESR1) is frequently overexpressed^18^ or mutated^19^ in breast carcinomas. The estrogen hormone binds to ERα, which activates transcription of estrogen-responsive genes^20^^,^^21^ leading to proliferation of mammary cells.^22^ Anti-estrogens are used as chemotherapeutic agents to counteract the proliferative effects of estrogen. However, ERα^-^ tumors do not respond to anti-estrogenic treatments and ERα^+^ tumors eventually become resistant to hormonal chemotherapies.

We have found that SETD6 associates with the NuRD complex subunits MTA2 and HDAC1. Consequently, SETD6 silences transcription of GAL4-dependent luciferase reporters. However, the expression of endogenous *PGR* and *TFF1* estrogen-responsive genes is repressed upon silencing of SETD6. The silencing of SETD6 expression culminates in cellular proliferation defects, apoptosis, and enhanced expression of the cell cycle inhibitor *CDKN1A*. Herein, we identify novel SETD6-associated proteins and depict SETD6 as a transcriptional regulator involved in nuclear receptor signaling. Importantly, the silencing of SETD6 induced proliferation defects independently of the status of ERα.

## Results

### SETD6 associates with chromatin proteins

SETD6 is composed of a SET catalytic domain, a substrate binding domain (SBD), and two putative LxxLL nuclear receptor interaction motifs (Fig. 1A). The first LxxLL motif is buried deep within the SET domain (Fig. S1). However, the second LxxLL motif found at the carboxy terminus of SETD6 is at the surface of the enzyme (Fig. S1) and quite possibly accessible for protein-protein interactions with nuclear receptors. SETD6 is ubiquitinated within the SBD at lysine 441,^23^ suggesting that substrate specificity could be regulated by post-translational modifications.

**Figure:**
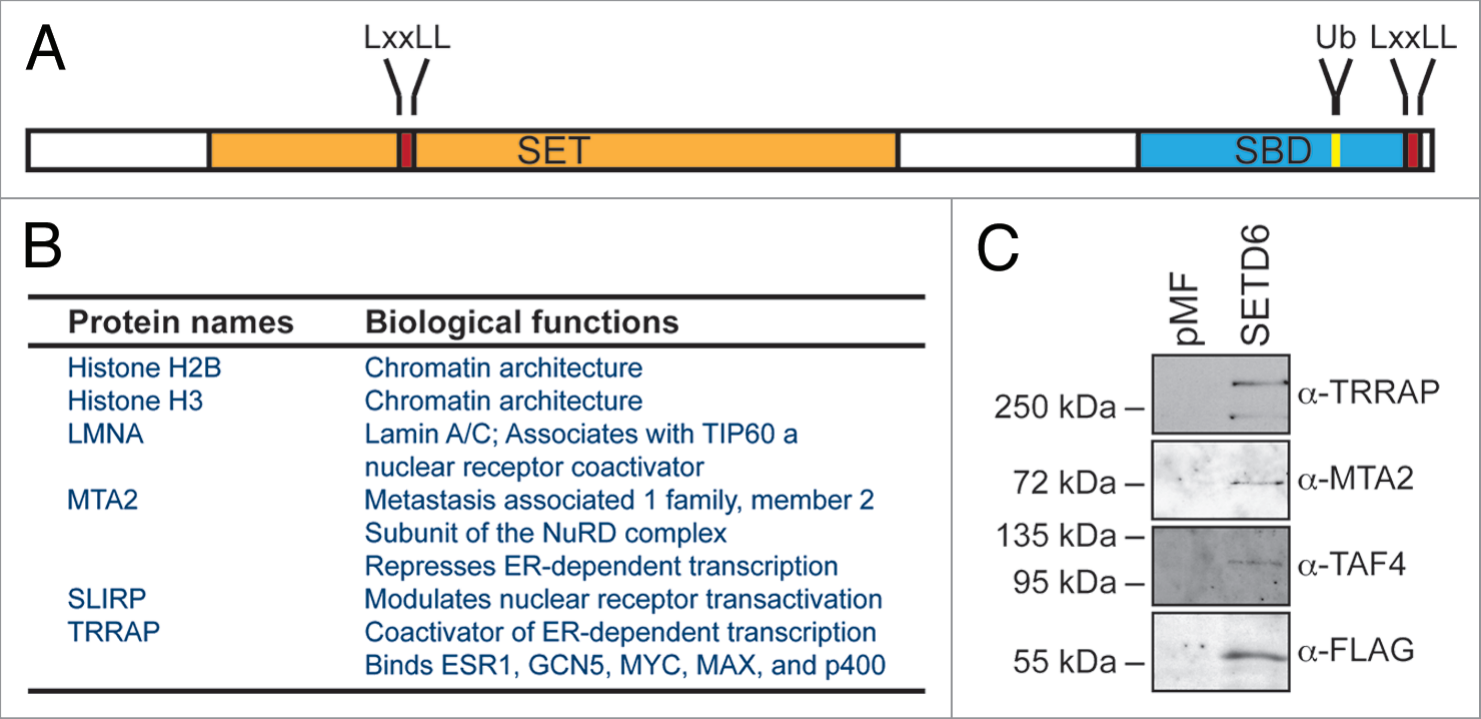
**Figure 1.** SETD6 associates with chromatin proteins. (**A**) Scaled graphical representation of SETD6. SETD6 contains a SET (Su[var]3–9, Enhancer-of-zeste, Trithorax) domain, substrate binding domain (SBD), two LxxLL nuclear receptor binding motifs, and is ubiquitinated at lysine 441 (Ub). (**B**) Table highlighting the nuclear receptor signaling factors identified by mass spectrometry that associate with SETD6. A complete detailed list of SETD6 associated proteins is available in the supplementary Table S2. (**C**) Validation of the mass spectrometric data by denaturing PAGE (SDS-PAGE) of α-FLAG M2-agarose immunoprecipitates followed by immunoblotting against the indicated endogenously expressed proteins.

Since little is known regarding SETD6 functions, we set out to identify SETD6-associated proteins. We have thus generated a stably-expressing FLAG-SETD6 cell line and a parental FLAG control cell line using the human osteosarcoma U2OS cell line. Those cells were transduced using pMSCV-FLAG (pMF) or pMF-SETD6 retroviral particles and then selected with puromycin. SETD6-associated peptides were affinity purified using an α-FLAG matrix. Then, SETD6-associated proteins were essentially identified by previously described mass spectrometric methods.^24^^,^^25^ Interestingly, several SETD6-associated proteins identified have known biological functions related to nuclear hormone signaling (Fig. 1B). Particularly interesting, several peptides from the nucleosome remodeling deacetylase (NuRD) complex subunit, MTA2,^26^ were identified (Table S1). A complete list of SETD6-associated proteins can be found in the online supplementary material section (Table S2). We then validated the mass spectrometric data by immunoblotting. An immunoprecipitation of FLAG-SETD6 followed by immunoblotting validated the association of SETD6 with endogenous MTA2, TAF4, and TRRAP (Fig. 1C). Exogenous expression of FLAG-SETD6 was extremely low and could only be detected once immunoaffinity purified (Fig. 1C).

### SETD6 associates with an MTA2/HDAC1 complex

Since the NuRD complex contains other subunits such as HDAC1, we investigated the association of SETD6 with various subunits of HDAC1-associated complexes. FLAG-tagged HDAC1 was co-expressed along with either YFP-tagged SETD6 or ING2, a known subunit of the SIN3A/HDAC1 complex. Although the expression of YFP-SETD6 was lesser than YFP-ING2, YFP-SETD6 co-immunoprecipitated with FLAG-HDAC1 to a greater extent than ING2 (Fig. 2A). These results suggest that SETD6 is an authentic HDAC1-binding protein.

**Figure:**
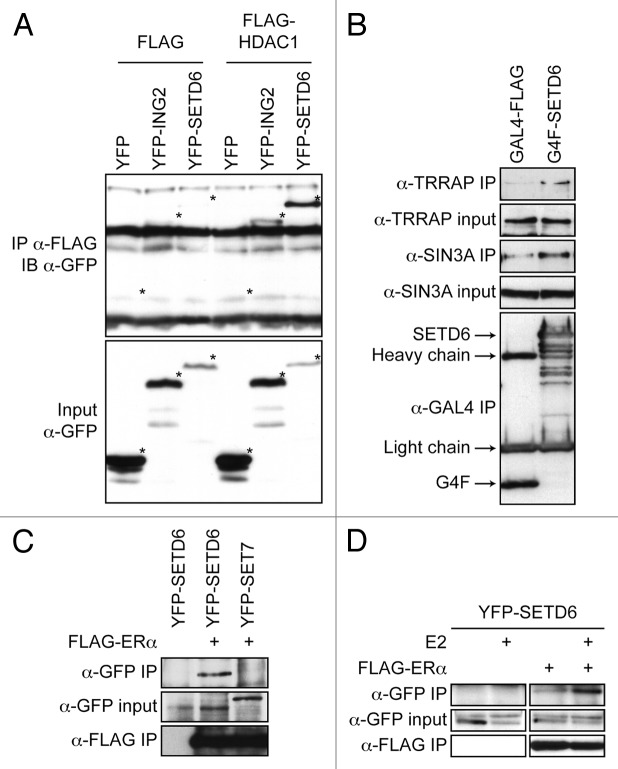
**Figure 2.** SETD6 associates with an MTA2/HDAC1 complex. (**A**) MCF7 cells were transiently transfected with either YFP alone, YFP-ING2, or YFP-SETD6 along with either empty FLAG vector or FLAG-HDAC1. The cells were lysed 48 h post-transfection and protein extracts applied on an α-FLAG M2-agarose matrix. The FLAG immunoprecipitates and 1/10 input were then analyzed by SDS-PAGE and immunoblotting with α-GFP antibody. An asterisk (*) in the bottom input panel indicates the position of each YFP-tagged proteins. The same asterisks were transposed in the IP panel to indicate that YFP on its own did not interact with HDAC1. (**B**) HEK293T cells were transiently transfected with either pcDNA3 GAL4-FLAG or GAL4-FLAG-SETD6. Protein extracts were immunoprecipitated as in panel **A** and then analyzed with the indicated antibodies. (**C**) MCF7 cells were transiently transfected with either empty vector or with FLAG-ERα and either YFP-SETD6 or YFP-SET7. α-FLAG immunoprecipitates were analyzed using α-GFP antibody. (**D**) MCF7 cells were transiently transfected with or without FLAG-ERα and YFP-SETD6. Estradiol (100nM) was added 44 h post-transfection and left for 4 h. The α-FLAG immunoprecipitates were analyzed with an α-GFP antibody

We then further characterized the SETD6/HDAC1 complex. FLAG-SETD6 was expressed in the highly transfectable HEK293T cell line and immunoaffinity purified. The immunoprecipitates were analyzed with antibodies directed against known SIN3A/HDAC1 complex subunits. As shown above, SETD6 associated with endogenous TRRAP (Fig. 2B). However, SETD6 failed to associate with SAP30 (a subunit of the SIN3A complex), SIN3A, RBAP46 (a subunit shared by both SIN3A and NuRD complexes), and CHD3 (MI-2 a subunit of the NuRD complex) (data not shown). Although the expression of *MTA2* and *TRRAP* at the mRNA level was mildly affected by the silencing of SETD6 (Fig. S2), the protein level remained unaffected (Fig. 2B).

Finally, since the NuRD complex is an important regulator of ER-dependent transcription^27^^,^^28^ and SETD6 contains 2 LxxLL nuclear receptor-binding motifs, we investigated the possible association between SETD6 and the nuclear receptor ERα. FLAG-tagged ERα was co-expressed in MCF7 cells with YFP-SETD6 or YFP-SET7 as a negative control. FLAG immunoprecipitates were analyzed by immunoblotting using an anti-GFP antibody (recognizes wild-type GFP, but also destabilized EGFP variants, such as EYFP) and ERα was found to specifically associate with SETD6 (Fig. 2C). Then, FLAG-ERα and YFP-SETD6 were co-expressed in MCF7 cells in the presence or absence of estradiol (E2). Interestingly, SETD6 association with ERα appeared to be enhanced in the presence of E2 (Fig. 2D). Finally, both LxxLL motifs found in SETD6 were converted to AxxAA and the YFP-SETD6_mLxxLL_ construct co-expressed with FLAG-tagged ERα. Interestingly, the SETD6_mLxxLL_ mutant retained its ability to associate with ERα (Fig. S3).

### SETD6 is a transcriptional regulator

To investigate the potential transcription regulatory activity of SETD6, we fused the methyltransferase to the DNA binding domain of GAL4 and used an heterologous GAL4-responsive *luciferase* reporter containing five GAL4 DNA-binding elements upstream of the SV40 promoter.^29^ In the absence of GAL4 DNA-binding elements in the reporter (G0), the SET domain alone (GAL4-SETD6_SET_) and the full-length protein (GAL4-SETD6_WT_) had only a minimal effect on the expression of *luciferase* from the reporter construct (Fig. 3A). However, in the presence of five GAL4 DNA-binding elements (G5), both GAL4-SETD6_SET_ and GAL4-SETD6_WT_ repressed transcription of the *luciferase* reporter by about 50 percent (Fig. 3A). Moreover, both GAL4-SETD6_SET_ and GAL4-SETD6_WT_ had a dose-responsive transcriptional effect on the G5TK*luciferase* reporter (Fig. 3B). We then compared the transcriptional repression activity of SETD6 to other KMTs, including the H3K27 methyltransferase EZH2 as well as the H3K9 methyltransferases SETDB1 and SUV39H2. Similarly to these KMTs involved in transcriptional silencing, SETD6 repressed the expression of the G5TK*luciferase* reporter (Fig. 3C). In addition, we compared SETD6 activity on the GAL4-responsive reporter to a known transcriptional activator, E2F1 (Fig. S4). The latter results validate our system by showing both silencing and activating responses. The GAL4-tagged constructs were expressed at equivalent level (Fig. 3).

**Figure:**
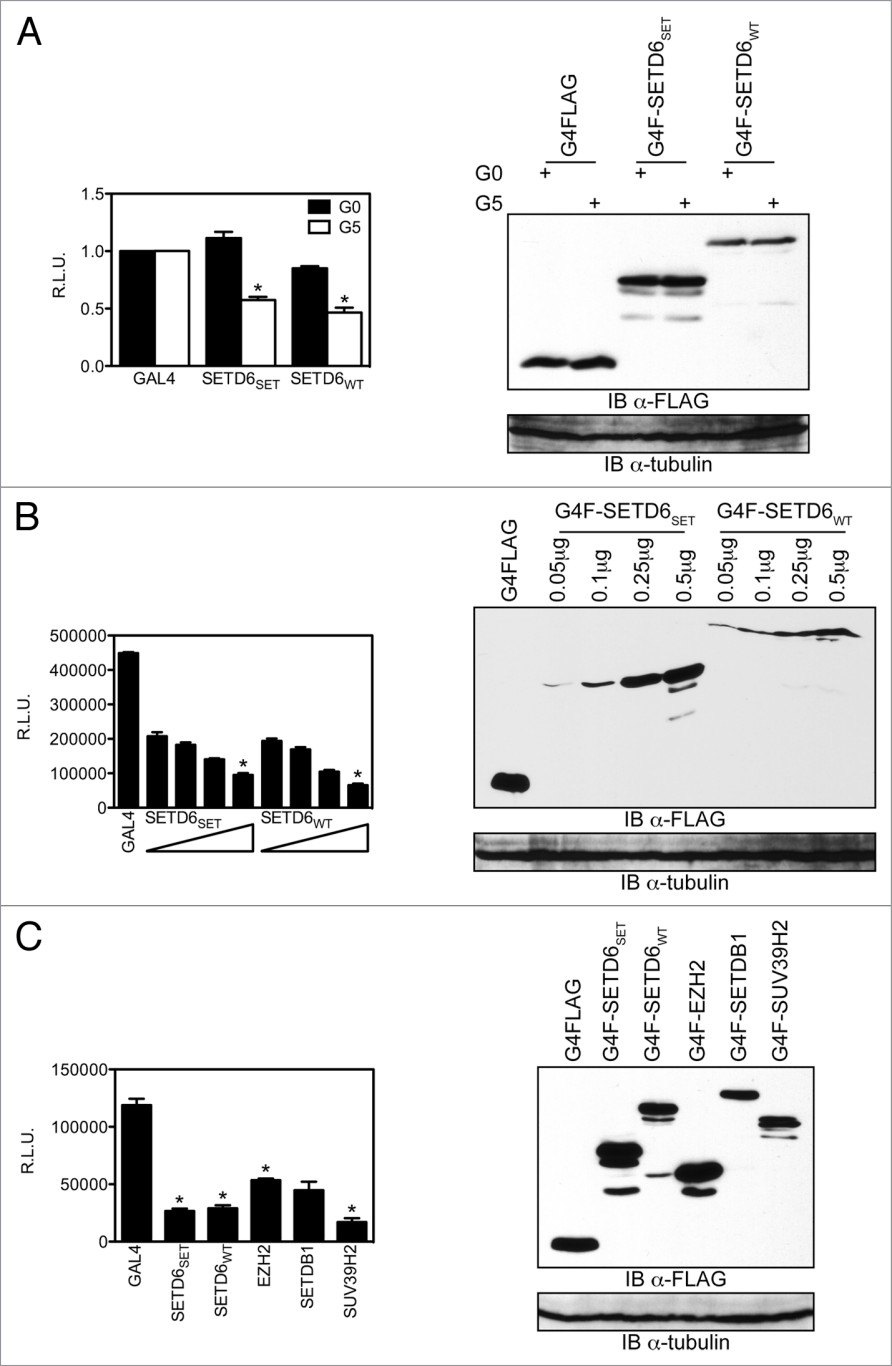
**Figure 3.** SETD6 is a transcriptional regulator. (**A**) HEK293 cells were seeded at a density of 100,000 cells per well of a 12-well plate and transfected the following day with 0.75μg of either pGL3 SV40*luc* (GAL4-unresponsive, i.e., G0) or pGL3 G5SV40*luc* (GAL4-responsive, i.e., G5) and 0.25μg GAL4_DBD_, GAL4-SETD6_SET_, or GAL4-SETD6_WT_. Values are expressed as relative luciferase units (R.L.U.) as measured 30 h post-transfection and error bars originate from the standard error of the mean from a representative experiment with each sample in quadruplets. *P values < 0.04. (**B**) As in panel **A**, but increasing amount (0.05 μg, 0.1 μg, 0.25 μg, and 0.5 μg) of GAL4-SETD6_SET_ or GAL4-SETD6_WT_ were transfected along with 0.5 μg reporter. *P values < 0.03 between the 0.0 5μg and 0.5 μg samples. (**C**) The indicated GAL4-tagged KMTs (0.25 μg) were transfected along with the G5SV40*luc* reporter (0.75 μg). *P values < 0.001. The expression level of each GAL4/FLAG-tagged constructs was assessed by denaturing PAGE of protein extracts followed by immunoblotting using the HRP-conjugated α-FLAG M2 or α-tubulin antibodies.

### SETD6 is a co-activator of estrogen-responsive genes

Since SETD6 associates with nuclear receptor signaling-associated factors and ERα, we investigated the expression of known estrogen-responsive genes by qPCR in control and SETD6-depleted breast carcinoma cell lines. The expression of SETD6 was silenced using previously validated shRNAs delivered by lentiviral transduction^16^ in human breast carcinoma MDA MB231 (ER^-^, PGR^-^ p53^mut^), MCF7 (ER^+^, PGR^+^ p53^wt^), and T47D (ER^+^, PGR^+^ p53^mut^) cell lines. The expression of the progesterone receptor gene *PGR* decreased substantially in all cell lines tested in response to SETD6-depletion (Fig. 4A–C). In the *TFF1*-expressing MCF7 cell line, the silencing of SETD6 also induced repression of this other estrogen-responsive gene (Fig. 4B). However, the expression of the estrogen-responsive gene *TGFA* remained unaltered in response to silencing of SETD6 expression in all three cell lines (Fig. 4A–C). The silencing of *SETD6* expression was recapitulated using siRNA and these also led to reduced expression of *TFF1* in MCF7 cells without altering the expression of *ESR1* (Fig. S5).

**Figure:**
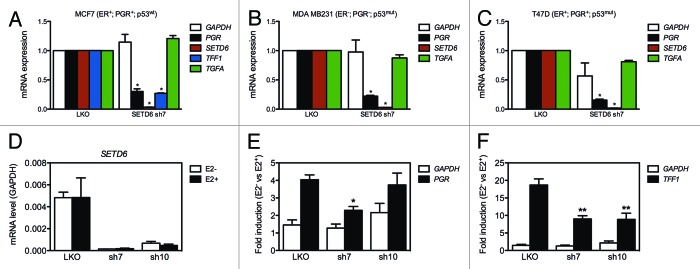
**Figure 4.** SETD6 is a co-activator of estrogen-responsive genes. (**A**) MDA MB231 cells were transduced with pLKO control shRNA or SETD6 shRNA (sh7) lentiviral particles. Total RNA was extracted 72 h post-transduction, reverse transcribed, and the expression of the indicated genes assessed by qPCR. The mRNA level from pLKO samples was set to 1. (**B**) As in panel **A**, but the experiment was conducted in MCF7 cells. (**C**) As in panel **A**, but the experiment was conducted in T47D cells. The * indicate p-values < 0.0006. (**D**) MCF7 were maintained in DMEM supplemented with charcoal-stripped serum prior to estradiol (E2) stimulation. The expression of *GAPDH*, *PGR*, *SETD6*, and *TFF1* was assessed by qPCR. * indicates p-values < 0.004 and ** < 0.03.

We then investigated the expression of *PGR* and *TFF1* in unstimulated (E2^-^) and estradiol (E2^+^) treated MCF7 cells. The induction of both genes was reduced by half in SETD6-silenced cells compared with control cells (Fig. 4D), further suggesting that SETD6 is playing a role in the expression of estrogen-responsive genes.

### SETD6 regulates proliferation of breast carcinoma cell lines

Nuclear receptor signaling generally regulates cellular proliferation. Moreover, activation of estrogen-responsive genes leads to increased proliferation of mammary cells.^22^^,^^30^ Since SETD6 associates with ERα and nuclear receptor signaling factors, and regulates estrogen-responsive genes, we investigated the impact of SETD6 silencing on cellular proliferation of human breast carcinoma cell lines. To address the potential role of SETD6 on proliferation, breast carcinoma cell lines were transduced with lentiviral particles that express shRNAs targeting *SETD6*. Loss of SETD6 expression led to proliferation defects as detected by a growth curve in the MCF7 cell line (Fig. S6). Specifically, all four SETD6-specific shRNA impaired proliferation rates when compared with the parental LKO control shRNA-expressing cells (Fig. S6). Proliferation rates were also assessed in a panel of human-derived breast carcinoma cell lines, including MDA MB231 (Fig. 5A), MCF7 (Fig. 5B), and T47D (Fig. 5C). Like MCF7 (Fig. 5B; Fig. S6), both ERα^-^ MDA MB231 and ERα^+^ T47D cell lines showed proliferation defects upon silencing of SETD6. Strikingly, SETD6 shRNA sh7 and sh10 cells displayed negligible growth over a period of 4 days (Fig. 5A–C). Moreover, silencing of SETD6 expression using siRNA instead of shRNA also reduced the proliferation of MCF7 and T47D cell lines (Fig. S7). These results demonstrate that SETD6 is required for normal cellular proliferation of human mammary cells.

**Figure:**
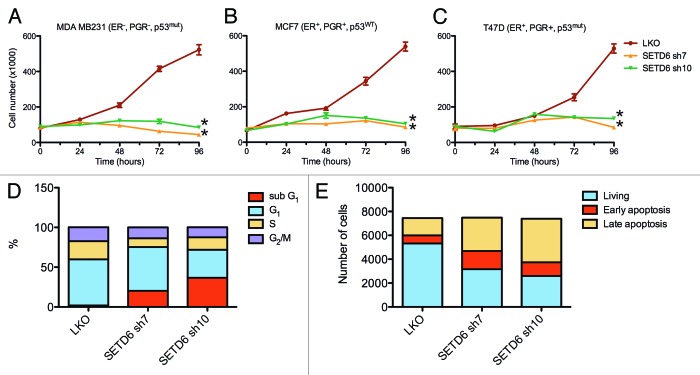
**Figure 5.** SETD6 regulates proliferation of breast carcinoma cell lines. (**A**) 50,000 MDA MB231 cells were transduced with pLKO control shRNA or SETD6 shRNA (sh7 or sh10) lentiviral particles. The next day, the media was replaced and cells were counted (Time = 0 h). The cells were then counted again every 24 h. (**B**) As in panel A, but the experiment was conducted in MCF7 cells. (**C**) As in panel **A**, but the experiment was conducted in T47D cells. Error bars originate from the standard error of the mean from a representative experiment with each sample counted four times. The * indicate p-values < 0.0004. (**D**) Cell cycle profile of MCF7 control cells (LKO) and SETD6-silenced cells (sh7 and sh10). (**E**) As in panel **D**, but the MCF7 cells were stained with Annexin V.

At late time points (72h and 96h) the number of cells had not changed appreciably compared with earlier time points in SETD6-silenced samples, suggesting that prolonged loss of SETD6 expression disturbed cell cycle progression. We thus investigated the expression of estrogen-responsive genes that are involved in cell cycle control. We observed that in MDA MB231, MCF7, and T47D cell lines the silencing of SETD6 systematically induced the expression of *CDKN1A* with a 6-fold increase of the cell cycle inhibitor in MDA MB231 (Fig. S8). Interestingly, the levels of *cyclin D1* (*CCND1*), *GREB1*, and *VEGFA* were lower in SETD6-silenced MCF7 cells (Fig. S8). Unlike the cell proliferation response to the silencing of SETD6, the transcriptional response seems to diverge between the cell lines, but *CDKN1A* was consistently upregulated in all cell lines.

To better understand the proliferation defects of SETD6-depleted cells, we then investigated the cell cycle profile of SETD6-silenced MCF7 cells by flow cytometry. The analysis revealed an increase in the sub-G_1_ population from about 2% in control cells (LKO) to 20–35% in SETD6-silenced cells (sh7 and sh10) (Fig. 5D), suggesting that decreased SETD6 expression induced apoptosis. Indeed, Annexin V staining, a marker of apoptosis, was enhanced by over 2-fold in SETD6-silenced MCF7 cells (Fig. 5E;Fig. S9).

## Discussion

Herein, we have identified by mass spectrometry and validated by immunoblotting the association of the methyltransferase SETD6 with several endogenous proteins that are involved in nuclear hormone receptor signaling. In particular, we found that SETD6 associates with MTA2 (Metastasis associated 1 family, member 2), a subunit of the NuRD complex^26^^,^^31^ that represses ER-dependent transcription.^32^^,^^33^ In addition, SETD6 associates with the RNA-binding protein SLIRP, which inactivates nuclear receptor-dependent transcription via an NCoR/HDAC complex.^34^ Interestingly, SETD6 represses transcription when it is artificially tethered to a promoter, in agreement with its association with NuRD complex subunits HDAC1 and MTA2. However, we observed that in the context of estrogen-responsive genes, SETD6 appears to be required for the expression of the estrogen-responsive genes *PGR* and *TFF1*, thus acting as a co-activator. In agreement with the repressive role of SETD6 in the GAL4 system and its association with HDAC1, the silencing of SETD6 led to increased expression of *CDKN1A*. Thus, it appears that depending on the transcriptional context, SETD6 can act either as a repressor or as an activator. Previous genome-wide studies of HDAC1 association with chromatin revealed that the deacetylase was bound along with RNA polymerase II to highly active promoters.^35^ In addition, the methyltransferase G9A, which methylates H3K9 and thus usually involved in transcriptional repression, was recently depicted as a necessary factor for the expression of estrogen-responsive genes.^36^ These studies highlight that some chromatin factors can have both a positive and a negative role on the regulation of gene expression.

Although SETD6 could associate with MTA2 and HDAC1, it failed to associate with other NuRD complex subunits (CHD3 and RBAP46) or with other SIN3A complex subunits (SAP30, SIN3A, and RBAP46). These results are not unexpected since the presence of SETD6 has never been detected in either biochemically purified NuRD or SIN3A complexes. Thus, SETD6 is either a substoichiometric component of the NuRD complex that could not be detected under previous experimental conditions or SETD6 is simply not part of the NuRD complex and associates only with MTA2/HDAC1. Further investigations will be necessary to elucidate the role of the association between SETD6 and MTA2/HDAC1.

In addition to its association with a transcription repressive HDAC1 complex, SETD6 associates with the known gene co-activators, LMNA, MGA, TAF4, and TRRAP. LMNA (Lamin A/C) associates with TIP60,^37^ a transcriptional co-activator of AR and ER.^38^^,^^39^ The association of SETD6 with LMNA may indicate a potential role for the methyltransferase in chromatin organization through the nuclear matrix. Both MGA and TAF4 are subunits of the MLL1/WDR5 histone H3 lysine 4 methyltransferase complex.^40^ TRRAP is a co-activator of ER-dependent transcription and associates with ERα, GCN5, MAX, MYC, and p400.^41^^,^^42^ Thus, the transcriptional effects observed on estrogen-responsive *PGR* and *TFF1* genes upon silencing of SETD6 expression, may reflect the association of SETD6 with these co-activators.

TAF4 is one of the 14 TATA-binding protein (TBP)-associated factors (TAF) composing the TFIID complex. Essentially, transcription is regulated by the binding of TBP to the TATA box and the nucleation of TAFs around TBP and the assembly of the pre-initiation complex (PIC), which is composed of RNA polymerase II and the general transcription factors TFIIA, TFIIB, TFIID, TFIIE, TFIIF, and TFIIH. TAF4 principal function appears to mediate interactions between TFIID and transcriptional activators such as SP1, CREB1, retinoic acid receptors, JUN, and basic helix-loop-helix E proteins family, which binds E boxes (CANNTG).^43^ Alternative splice variants of TAF4 lack parts of the TAF homology domain, which mediates association with a variety of transcriptional factors. These TAF4 variants are differentially expressed in adult tissues, stem cells, and differentiated cells, potentially leading to different tissue-specific gene expression patterns.^44^ To investigate how SETD6 regulates the expression of estrogen-responsive genes, we have performed chromatin immunoprecipitations of stably-expressed FLAG-SETD6 and transiently overexpressed FLAG-SETD6 in MCF7 cells, but failed to detect its presence at the promoter of *CDKN1A*, *PGR*, or *TFF1* (Fig. S10). Specifically, the association of SETD6 with chromatin was investigated at -1000 and -500 base pairs upstream of the transcriptional start site and at the transcriptional start site of *CDKN1A*, *PGR*, and *TFF1*. Although not evidence of absence, the absence of evidence for the presence of SETD6 at regulatory elements of *CDKN1A*, *PGR*, and *TFF1* suggests that the silencing of SETD6 may affect indirectly the expression of those genes. The association of SETD6 with TAF4 may alter the activity of the TFIID complex with consequences on the transcription of genes such as *CDKN1A*, *PGR*, and *TFF1*, irrespectively of ER status.

The transformation and transcription domain-associated protein (TRRAP) was originally identified as an E2F1 and MYC-associated protein.^45^ TRRAP also associates with the nuclear receptor ERα,^46^ and numerous histone acetyltransferase (HAT) complexes.^47^ Through its association with various HAT complexes, TRRAP is involved in most DNA transaction processes, including transcription, replication, and repair. Interestingly, TRRAP^−/−^ cells have impaired clonogenicity and proliferation,^48^ similarly to SETD6-silenced mouse embryonic stem cells.^16^ Recently, TRRAP was rediscovered in a screen to identify synthetic interactions with MYC overexpression.^49^ Interestingly, SETD6 was originally found to associate with the ATPases RUVBL1 and RUVBL2,^50^ which are found in the human TIP60 histone acetyltransferase complex with TRRAP^51^ and involved in histone exchange of H2A variants.^52^ Thus, it could be hypothesized that the interaction between SETD6 and TRRAP, RUVBL1, or RUVBL2 may affect the deposition of H2AZ at promoters, including *CDKN1A*, *PGR*, and *TFF1*, to regulate the expression of estrogen-responsive genes irrespectively of ER status. Indeed, silencing of SETD6 expression led to reduced level of the histone variant H2AZ and its methylated H2AZK7^me1^ form at the enhancer and promoter of *TFF1* (Fig. S11).

The association of SETD6 with nuclear hormone receptor signaling elements, ERα and MTA2 in particular, and the misregulation of estrogen-responsive genes in SETD6-depleted cells, prompted us to investigate the proliferation of those cells. All breast carcinoma cell lines tested stopped proliferating upon silencing the expression of SETD6. Importantly, both ER^+^ (MCF7 and T47D) and ER^-^ (MDA MB231) cells were similarly affected by the silencing of SETD6 expression. In agreement with the cell blockage induced by the silencing of SETD6, all breast carcinoma cell lines investigated expressed increased levels of *CDKN1A* in response to reduced expression of SETD6. The effect of silencing SETD6 on proliferation was further investigated in MCF7 and we observed an increase in sub-G_1_ population and Annexin V staining, demonstrating that silencing of SETD6 induces apoptosis.

Finally, we have found that the regulation of estradiol-responsive genes and cellular proliferation by SETD6 is independent of ERα status. Since ERα^-^ tumors do not respond to anti-estrogenic treatments and ERα^+^ tumors eventually become resistant to hormonal chemotherapies, the regulation of ER signaling by SETD6 independently of ERα status could potentially be exploited in future therapies. Thus, these results may have profound implication in the treatment of breast cancer patients.

## Materials and Methods

### Plasmids

The cDNA of SETD6 was purchased from OpenBiosystems and inserted in pMSCVpuro (Clontech), pcDNA3 GAL4-FLAG, and pcDNA3 YFP using restriction endonucleases (NEB). The FLAG-HDAC1 construct was described previously.^16^^,^^53^ The pLKO shRNA constructs were obtained from OpenBiosystems and described previously.^16^

### Antibodies

α-TRRAP (ab73546), α-SIN3A (ab129087), α-SAP30 (ab65559), TIP49A (ab133513), and α-RBAP46 (ab109285) were purchased from Abcam, α-FLAG M2-agarose and HRP-conjugated α-FLAG M2 (A8592) were obtained from Sigma, and α-GFP (632375) from Clontech.

### Immunoprecipitations

Cells were lysed in lysis buffer (50 mM Tris-HCl pH 7.5, 100 mM NaCl, 0.5% Triton X-100, 10% glycerol, and EDTA-free protease inhibitors cocktail from Roche). Cleared lysates were inverted overnight at 4 °C with 10μL of α-FLAG M2-agarose matrix (Sigma, A2220). The next day, the agarose beads were washed 4 times with 1mL of lysis buffer. Bound proteins were resolved by PAGE, transferred onto PVDF membranes, and analyzed by immunoblotting.

### Growth curves

Cells were seeded at 50 000 cells per well of a 6-well plate for each time point. Approximately 6 h later, when the cells had adhered, the cells were transduced with shRNA-expressing lentiviral particles. The following day (time 0h), the media was changed and the first plate counted using an hematocymeter. The same counting procedure was repeated at 24 h, 48 h, and 96 h, while an extra plate was kept for RNA extraction and gene expression analysis.

### Flow cytometry analysis

Cell cycle was assessed by suspending MCF7 cells in a propidium iodide staining cocktail (0.8% Triton X-100, 50 μg/mL propidium iodide, and 75 μg/mL RNase A) for 10 min at room temperature. Apoptosis was assessed by Annexin V Alexa Fluor488 and propidium iodide staining (Life Technologies, V13241). Stained cells were immediately injected into a BD FACSCalibur where 10 000 cells were evaluated per sample.

### Mass spectrometric analysis

SETD6-bound proteins were immunoaffinity purified as described previously.^25^ Then, the SETD6-bound proteins were analyzed and identified by mass spectrometry as described elsewhere.^24^

### Knockdowns and qPCR

Duplex siRNA were obtained from Dharmacon (cat. # MQ-014486–00–0005) and shRNA constructs were obtained from OpenBiosystems (RHS4533-NM_024860). Gene expression was assessed by qPCR on reverse transcribed total RNA. Briefly, total RNA was extracted using TRIzol reagent (Invitrogen, 15596–026), 2.5 μg of total RNA was reverse transcribed using SuperScript VILO (Invitrogen, 11755–050), and qPCR performed using QuantiTect SYBR Green (QIAGEN, 204143) on an ABI PRISM 7900HT Sequence Detection System (Applied Biosystems). Sequence of gene-specific primers used for qPCR are available in the supplementary section (Table S3).

## Supplementary Material

Additional material

## Notes

10.4161/epi.28864
