# Supplementary material for: SETD6 controls the expression of estrogen-responsive genes and proliferation of breast carcinoma cells
Source: Epigenetics. 2014 Apr 21;9(7):942–50. doi: 10.4161/epi.28864 (PMC4143409; doi:10.4161/epi.28864)
Supplement: Additional material [file epi-9-942-s01.pdf]

Landes Bioscience www.landesbioscience.com

Supplemental Material to:

Daniel J O'Neill, Stuart Charles Williamson, Dhuha Alkharaif, Isabella Christina Mazzaro Monteiro, Marilyn Goudreault, Luke Gaughan, Craig N Robson, Anne-Claude Gingras, and Olivier Binda

SETD6 controls the expression of estrogen-responsive genes and proliferation of breast carcinoma cells

Epigenetics 2014; 9(7)
http://dx.doi.org/10.4161/epi.28864

http://www.landesbioscience.com/journals/epigenetics/article/28864/

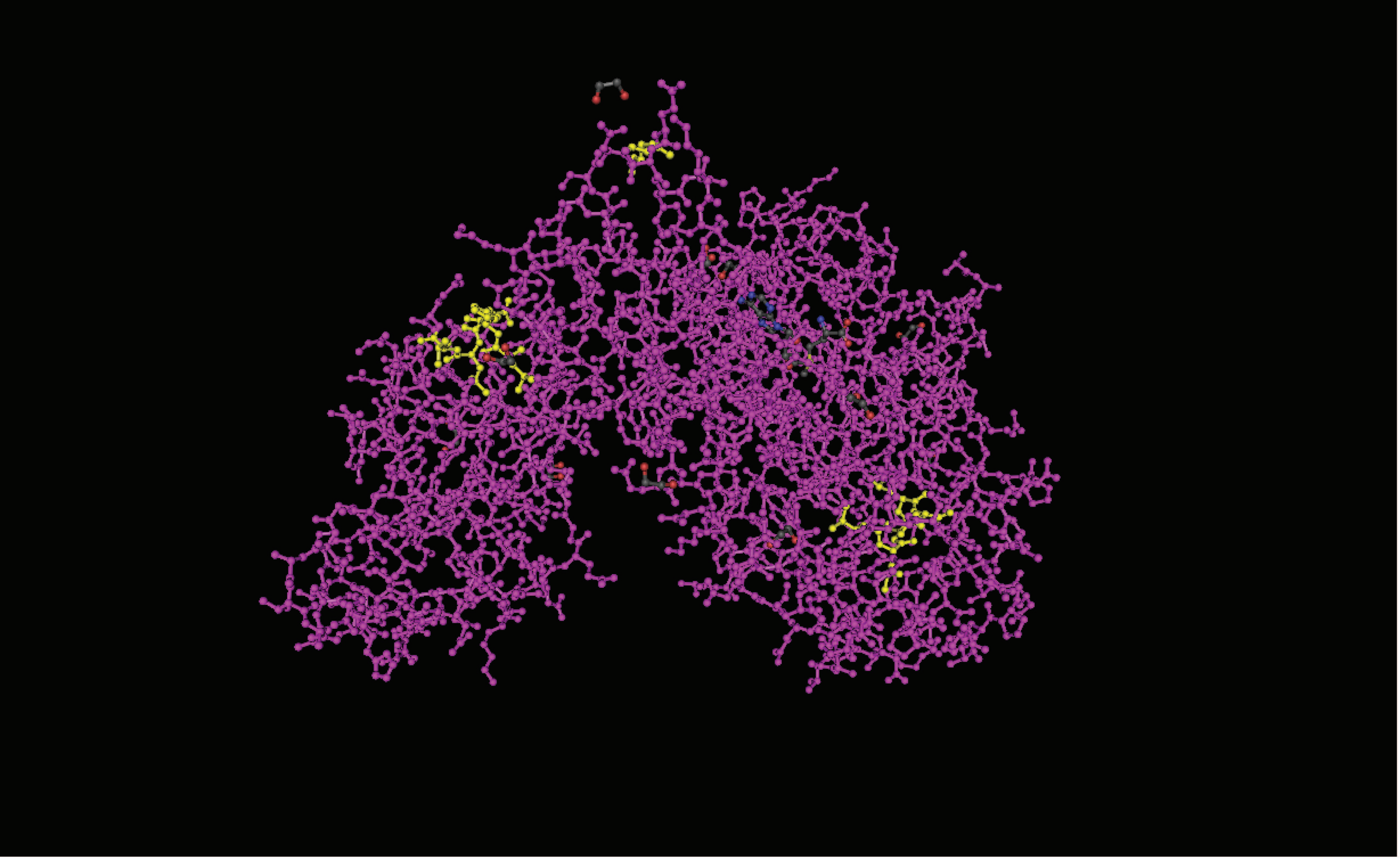

Supplementary Figure S1: Crystal structure of SETD6 (PDB 3RCO). The first LxxLL (right; highlighted yellow) motif is buried within the catalytic domain. The second LxxLL motif (left; highlighted yellow) is at the surface of SETD6. Lysine 441, which can be ubiquitinated is also highlighted (top; yellow).

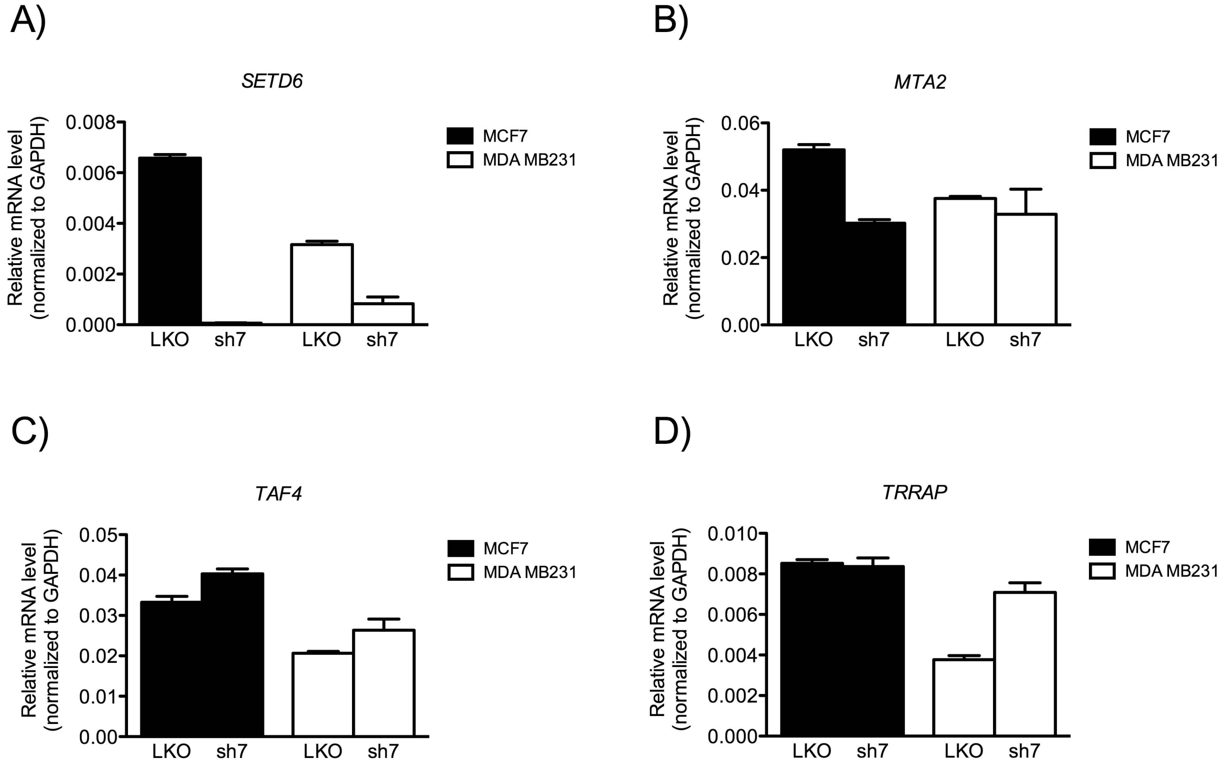

Supplementary Figure S2: SETD6 expression was silenced in either MCF7 or MDA MB231 and the expression of SETD6 and SETD6-associated proteins analysed by real-time qPCR using gene-specific primer sets.

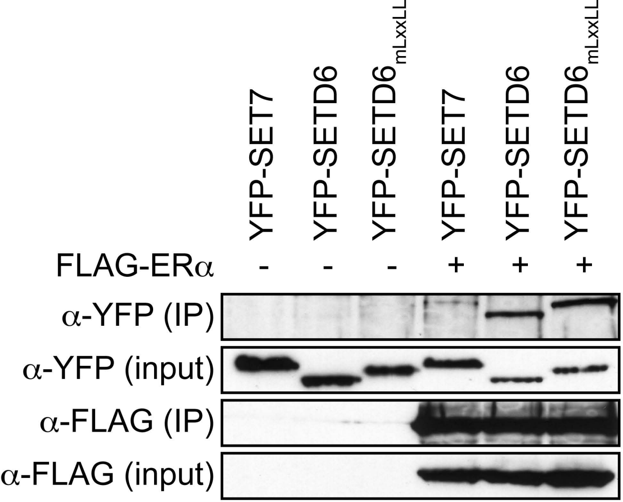

Supplementary Figure S3: HEK293T cells were transfected with or without FLAG-ERα along with YFP-SET7, YFP-SETD6, or YFP-SETD6$_{mLxxLL}$. Cell lysates were immunoprecipitated using α-FLAG M2 agarose. The immunoprecipitates were analysed by immunoblotting using the indicated antibodies.

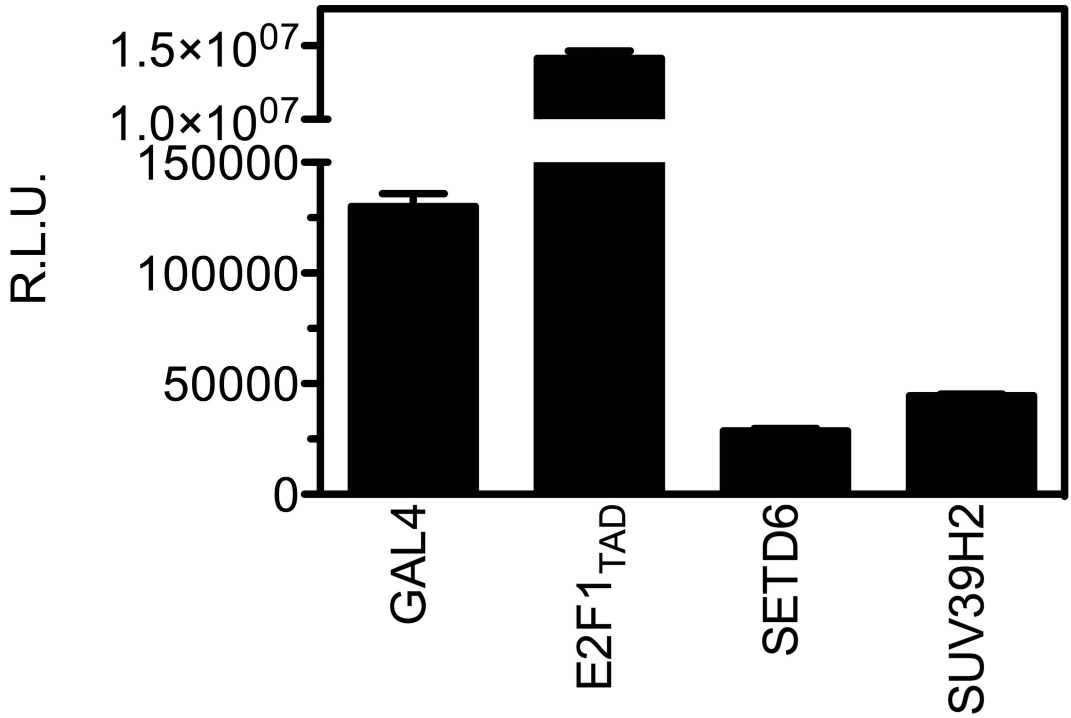

Supplementary Figure S4: HEK293T cells were transfected with pG5TKluc reporter and the indicated GAL4-tagged proteins. E2F1 transactivation domain (TAD) was used as a transcriptional activation control.

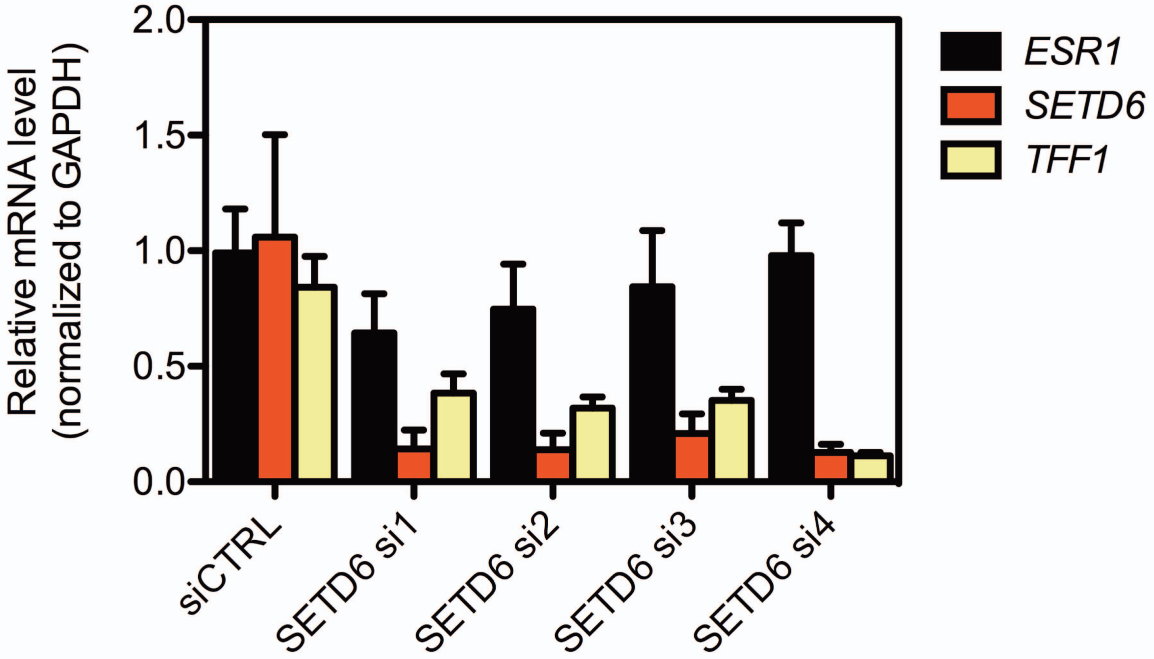

Supplementary Figure S5: MCF7 cells were transfected with either non-specific siRNA control or SETD6-specific siRNA (si1-si4). Expression of the indicated genes was assessed by qPCR from reverse transcribed total RNA extracted from cells.

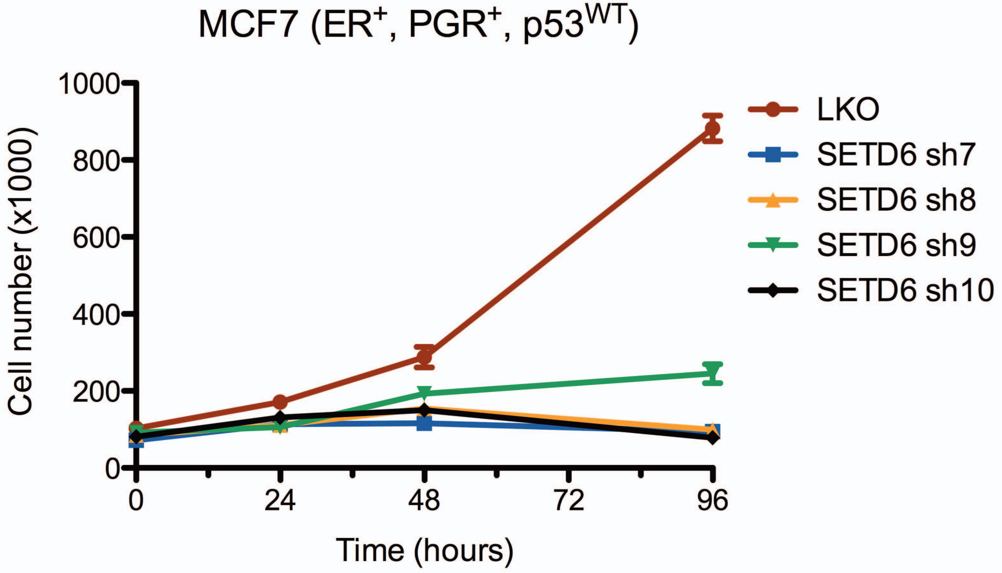

Supplementary Figure S6: MCF7 cells were counted and 50,000 cells were plated per well of 5 6-well plates. Approximately 5-6 hours later (when the cells had adhered to the plastic), the cells were transduced with the indicated shRNA-expressing lentiviral particules. The next day, the media was replaced (and lentiviral particules removed) and the cells from one plate counted in triplicate (time 0 hour). The counting was repeated at 24, 48, and 96 hours. Total RNA was extracted from the fifth plate at time 72 hours to assess the knockdown efficiency.

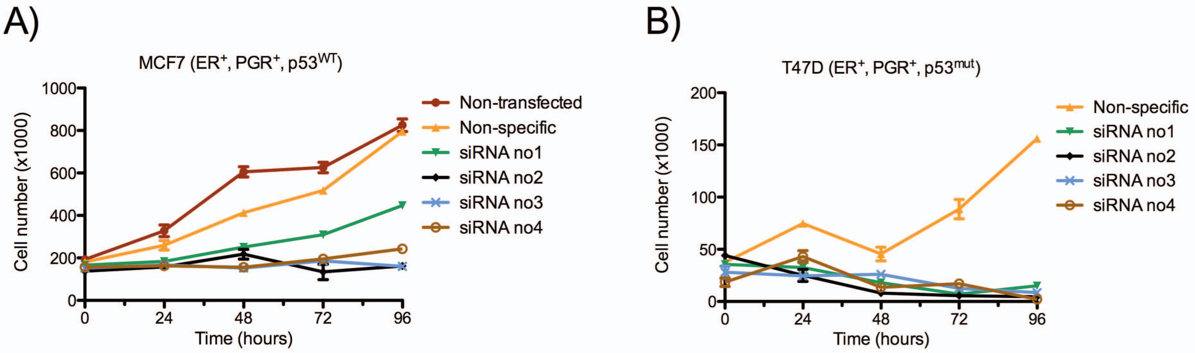

Supplementary Figure S7: MCF7 cells (**A**) or T47D (**B**) were counted and 50,000 cells per well of six 6-well plates were reverse transfected with the indicated siRNA particules. Two days later, the cells from one plate were counted in triplicate (time 0 hour). The counting was repeated at 24, 48, 72, and 96 hours. Total RNA was extracted from the fifth plate at time 72 hours to assess the knockdown efficiency (see **Figure S3** for qPCR results).

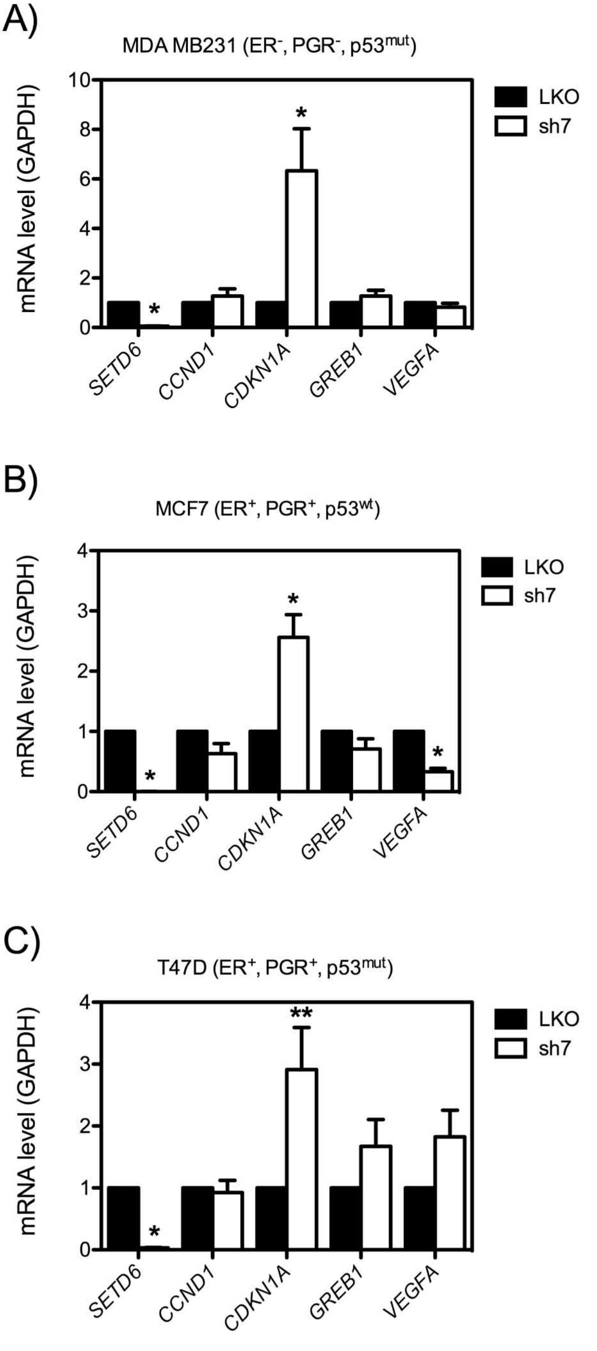

Supplementary Figure S8: Total RNA was extracted from control and SETD6-silenced cells. The expression of indicated genes was assessed by qPCR. * indicate p-values < 0.025 and ** < 0.06.

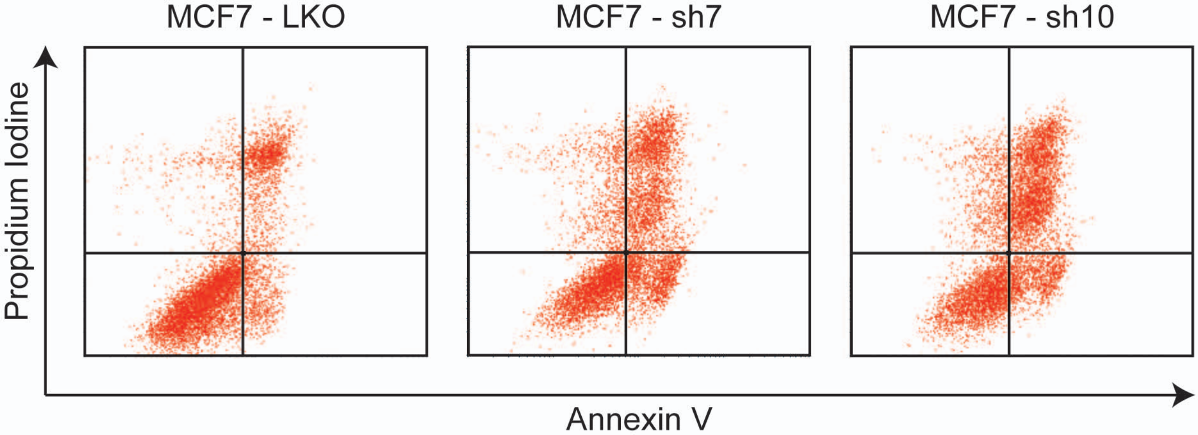

Supplementary Figure S9: MCF7 control cells (LKO) and SETD6-silenced cells (sh7 and sh10) were stained with propidium iodine and Annexin V and analysed by FACS. Annexin V^+/PI^- (lower right quadrant) were considered as early apoptosic cells, while Annexin V^+/PI^+ (upper right quadrant) were considered as late apoptosic cells.

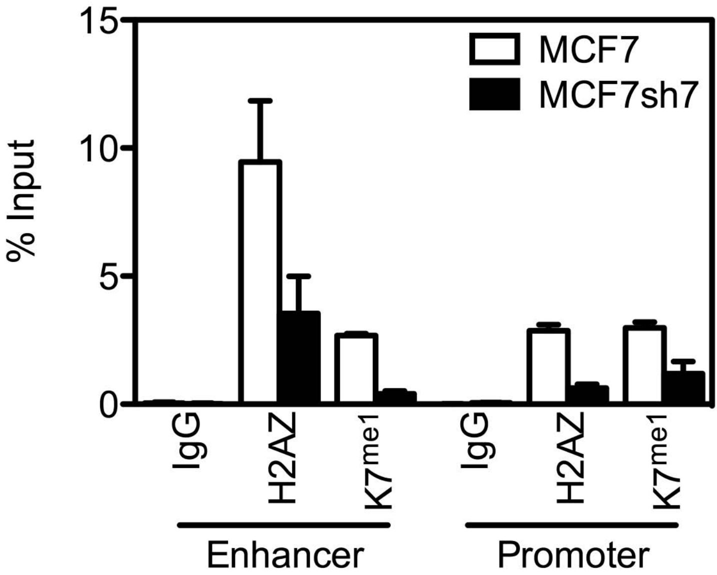

Supplementary Figure 11: Chromatin immunoprecipitation of the histone variant H2AZ and its lysine methylated form at the enhancer and promoter regions of the *TFF1* gene.

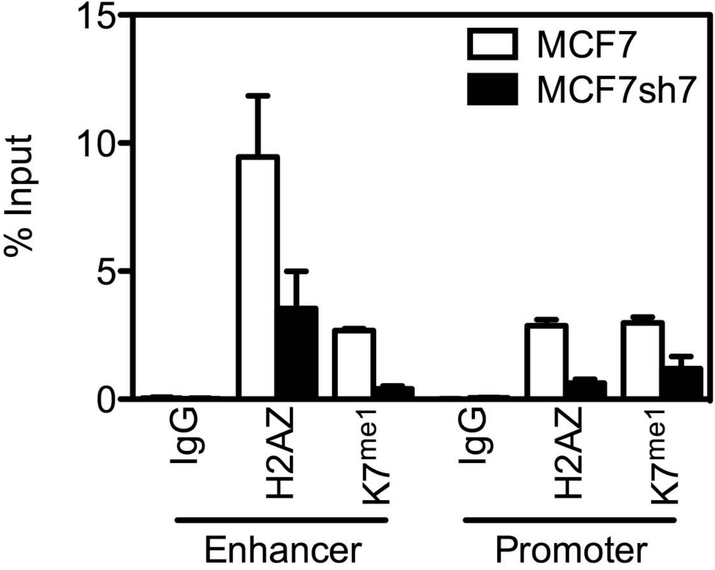

Supplementary Figure 11: Chromatin immunoprecipitation of the histone variant H2AZ and its lysine methylated form at the enhancer and promoter regions of the *TFF1* gene.

Table S1: Sequence of the SETD6-bound peptides.

SETD6-binding proteins	Peptide Sequence	Region
MGA	GFDNPEENSSEFPVTFK	2119-2135
	VLQSEGEAVDPEANVIK	1993-2009
MTA2	DISSSLNSLADSNAR	50-64
	LNPADAPNPVVFVATK	596-611
TAF4	FFEQLDQIEK	946-955
	ALSAVSAQAAAAQK	804-817
	EMQQQELAQMR	990-1000
TRRAP	TALRPDMWPK	2114-2123
	EVQSNMVPRSMLK	3611-3623
	FMPRVEIVQK	3470-3479

Table S2: Proteins identified specifically from the FLAG-SETD6 pulldowns.

Hit Gene Name	Hit Gene ID	Hit Protein ID	Hit Score	Peptide Number	Unique Peptide Number	Coverage	Frequency	Shared Frequency
ACADVL	37	4557235	106	4	3	7.5	5.78	50
ACO2	50	4501867	91	2	2	4	1.7	25
AK2	204	4502013	54	2	1	10.5	7.14	50
C14orf156	81892	13654278	69	3	2	22.9	18.03	25
CD44	960	48255935	48	3	1	1.6	3.06	25
CDC42	998	4757952	45	2	1	6.8	7.48	25
CNOT3	4849	7657387	59	1	1	4.6	2.72	25
COX4I1	1327	4502981	70	3	2	13	12.24	25
COX5A	9377	190885499	93	3	2	26	16.33	50
COX5A	9377	190885499	66	2	2	20.7	16.33	50
DAP3	7818	4758118	119	3	2	7.3	18.71	25
DLST	1743	19923748	63	2	2	7.3	11.9	25
EIF4A3	9775	7661920	68	2	1	3.2	17.69	25
ESYT1	23344	14149680	232	6	6	8.7	15.31	25
FAM98A	25940	56699482	59	3	1	6.2	10.54	25
FH	2271	19743875	65	2	2	8	3.74	50
GARS	2617	116805340	52	2	1	3.9	9.52	25
GFAP	2670	196115290	64	2	1	4.9	19.05	25
GLS	2744	156104878	53	2	1	3.1	2.04	25
GPC1	2817	167001141	88	2	2	8.1	1.7	25
HIST1H3F	8968	4504281	65	2	1	23.5	15.31	50
HIST1H3F	8968	4504281	56	4	1	23.5	15.31	50
HIST2H2BE	8349	4504277	189	16	1	27	11.9	25
HIST2H3D	653604	31742503	97	1	1	23.5	8.16	25
HSPB1	3315	4504517	184	8	4	36.1	9.18	50
HSPB1	3315	4504517	93	3	2	8.8	9.18	50
HSPE1	3336	4504523	101	3	2	25.5	15.31	50
ITGB1	3688	19743813	75	3	1	2.6	3.06	25
KIF4A	24137	116686122	58	3	1	2.3	8.84	25
KRT222	125113	22748757	66	5	1	7.1	6.46	25
KRT80	144501	125628632	55	2	1	5.7	17.01	25
KTN1	3895	33620775	86	4	3	3.5	12.59	25
LMNA	4000	5031875	558	22	12	28.8	14.63	100
MGA	23269	256017159	146	4	2	1.2	18.37	25
MRE11A	4361	5031923	56	2	2	2.7	8.5	25
MRPS6	64968	16554616	50	2	1	19.2	3.4	25
MTA2	9219	14141170	58	2	2	4.6	11.22	25
PLOD1	5351	32307144	70	2	2	5.1	8.84	25
PPP1R9B	84687	140972063	78	5	1	3.8	18.71	25
RAB10	10890	256222019	58	2	1	5.5	5.78	25
RAB11A	8766	4758984	57	3	2	18.5	6.8	25
RAB13	5872	4506363	83	3	2	11.8	1.36	25
RAB1B	81876	13569962	112	5	2	16.4	7.82	25
RALY	22913	8051631	66	3	2	6.2	9.52	25
RBM27	54439	168229174	62	2	2	3.5	13.95	25
S100A10	6281	4506761	78	4	2	35.1	3.4	75
S100A10	6281	4506761	86	4	2	35.1	3.4	75
S100A11	6282	5032057	47	1	1	15.2	1.7	25
SETD6	79918	238550105	610	25	11	44.8	0.34	50
SETD6	79918	238550105	209	6	4	17.8	0.34	50
SFXN1	94081	23618867	61	2	2	7.1	6.8	25
SLC3A2	6520	61744477	115	4	3	8.4	11.22	25
STBD1	8987	4503977	73	2	2	7.8	2.04	25
STOML2	30968	7305503	63	2	2	13.2	19.39	50
STON2	85439	21361863	60	4	1	1	3.4	25
TAF4	6874	110832843	124	4	3	3.2	13.95	25
TMEM33	55161	224589127	80	2	2	8.5	6.12	25
TRRAP	8295	4507691	79	3	2	0.9	13.95	25
VDAC3	7419	25188179	57	2	1	7.4	12.59	25

Table S3: Sequence of the primer sets used for qPCR. The primers were selected from the NCBI Nucleotide database using the utility Primer-BLAST. The primers used for gene expression (first section) were designed to span exon-exon junctions and be separated by at least one intron when possible. The primers in the second section were used for the ChIP assays.

**Gene**		**Primer set 1**	**Primer set 2**
CDKN1A	Fwd	CAGCTGCCGAAGTCAGTTCC	
	Rev	GTTCTGACATGGCGCCTCCT	
CCND1	Fwd	GCCCTCGGTGTCCTACTTCAAA	
	Rev	TTCTGTTCCTCGCAGACCTCC	
GAPDH	Fwd	CGGAGTCAACGGATTTGGTCG	GTTCGACAGTCAGCCGCATC
	Rev	ACGGTGCCATGGAATTTGCC	CGACCAAATCCGTTGACTCCG
GREB1	Fwd	GGATGAGGAGCTGGGGACAG	
	Rev	GCTGAACCGGAAGCCTTGGA	
MTA2	Fwd	ACGGGGGAAATGCAGTGTGA	
	Rev	ACGGGGTCAAACACCAGTGA	
PGR	Fwd	GCAATGGAAGGGCAGCACAA	
	Rev	TCGACCTCCAAGGACCATGC	
SETD6	Fwd	GTGGCCCTTGTGATGGCCTA	TGACAGTTCGTGAGGCAGCA
	Rev	ACCATCCGAAGACAATTCGCAG	GCAGGCATGCACAGTACCTTT
TAF4	Fwd	AGGGCAGCAAAGTCTCGGTC	
	Rev	GCGTCCCGCTGTCTCATTTG	
TGFA	Fwd	AAAATGGTCCCCTCGGCTGG	
	Rev	GGGTCTGCACTCAGCGGG	
TFF1	Fwd	TCGCCTTTGGAGCAGAGAGG	
	Rev	ACACGTCTCTGTCTGGGCCT	
TRRAP	Fwd	ACGACTTGTCCCACTGGAGC	
	Rev	ACAATCGATGATGAATGCATGCCG	
VEGFA	Fwd	CACAACAAATGTGAATGCAGACCAA	
	Rev	TTGCGCTTTCGTTTTTGCCC	
CDKN1A TSS	Fwd	ctgtggctctgattggctttct	
	Rev	gacaaaatagccaccagcctct	
CDKN1A -500	Fwd	ggagactgcagtgagctgagat	
	Rev	ccctggctttttgttttcattt	
CDKN1A -1000	Fwd	atgatctcagctcactgcaacc	
	Rev	ggcacctgtagtcccaggtatt	
TFF1 TSS	Fwd	gcaaacagagcctgccctataa	
	Rev	gcctcctctctgctccaaag	
TFF1 -500	Fwd	accatgcctggctaatttttgt	
	Rev	aggcggatcacttaaagtcagg	
TFF1 -1000	Fwd	cagggaggagtgcaatgaataa	
	Rev	cctgctcggagctttctcttt	
PGR TSS	Fwd	cctagaggaggaggcgttgtta	
	Rev	ccacacgcacaaatacaacaag	
PGR -500	Fwd	cccactcccaaatttctttgtc	
	Rev	tgctttcaagtttctcctgctg	
PGR -1000	Fwd	atatagcccctccccagatgat	
	Rev	aatgcagaagactccagaaggtg	
